# Vinpocetine Reduces Carrageenan-Induced Inflammatory Hyperalgesia in Mice by Inhibiting Oxidative Stress, Cytokine Production and NF-κB Activation in the Paw and Spinal Cord

**DOI:** 10.1371/journal.pone.0118942

**Published:** 2015-03-30

**Authors:** Kenji W. Ruiz-Miyazawa, Ana C. Zarpelon, Felipe A. Pinho-Ribeiro, Gabriela F. Pavão-de-Souza, Rubia Casagrande, Waldiceu A. Verri

**Affiliations:** 1 Departamento de Ciências Patológicas, Universidade Estadual de Londrina, Londrina, Paraná, Brasil; 2 Departamento de Ciências Farmacêuticas, Universidade Estadual de Londrina Londrina, Hospital Universitário, Londrina, Paraná, Brasil; Temple University, UNITED STATES

## Abstract

Vinpocetine is a safe nootropic agent used for neurological and cerebrovascular diseases. The anti-inflammatory activity of vinpocetine has been shown in cell based assays and animal models, leading to suggestions as to its utility in analgesia. However, the mechanisms regarding its efficacy in inflammatory pain treatment are still not completely understood. Herein, the analgesic effect of vinpocetine and its anti-inflammatory and antioxidant mechanisms were addressed in murine inflammatory pain models. Firstly, we investigated the protective effects of vinpocetine in overt pain-like behavior induced by acetic acid, phenyl-p-benzoquinone (PBQ) and formalin. The intraplantar injection of carrageenan was then used to induce inflammatory hyperalgesia. Mechanical and thermal hyperalgesia were evaluated using the electronic von Frey and the hot plate tests, respectively, with neutrophil recruitment to the paw assessed by a myeloperoxidase activity assay. A number of factors were assessed, both peripherally and in the spinal cord, including: antioxidant capacity, reduced glutathione (GSH) levels, superoxide anion, tumor necrosis factor alpha (TNF-α) and interleukin 1 beta (IL-1β) levels, as well as nuclear factor kappa B (NF-κB) activation. Vinpocetine inhibited the overt pain-like behavior induced by acetic acid, PBQ and formalin (at both phases), as well as the carrageenan-induced mechanical and thermal hyperalgesia and associated neutrophil recruitment. Both peripherally and in the spinal cord, vinpocetine also inhibited: antioxidant capacity and GSH depletion; increased superoxide anion; IL-1β and TNF-α levels; and NF-κB activation. As such, vinpocetine significantly reduces inflammatory pain by targeting oxidative stress, cytokine production and NF-κB activation at both peripheral and spinal cord levels.

## Introduction

Modern lifestyle has raised life expectancy, but also increased the incidence of chronic diseases [1], consequently increasing chronic pharmaceutical usage. Inflammation is a hallmark of many chronic diseases, with the long-term use of steroids and/or non-steroid anti-inflammatory drugs leading, in many instances, to hormonal side effects and gastric lesions [2,3]. As such, the development of novel anti-inflammatory drugs and treatment approaches is urgently needed.

Vinpocetine (ethyl apovincamine-22-oate) is a synthetic ethyl ester of the alkaloid apovincamine, which is isolated from the leaves of *Vinca minor*, commonly known as the lesser periwinkle [4]. Vinpocetine has been used in the management of various cerebrovascular disorders, including cerebral infarction and residual cerebral hemorrhage [5], as well as in the treatment of cognitive disorders [4]. Importantly, no significant vinpocetine side effects have been reported [6], whilst its positive effects on cognition are even apparent in health volunteers [7].

As well as being a phosphodiasterase-1 inhibitor [8], the emerging literature suggests that vinpocetine also has potent anti-inflammatory effects via phosphodiasterase-1-independent inhibition of nuclear factor kappa B (NF-κB) signaling and the production of pro-inflammatory cytokines, such as IL-1β and TNF-α [9]. Moreover, it has been shown that vinpocetine inhibits neuronal reactive oxygen species (ROS) production [10,11], thereby decreasing oxidative stress [12]. The pro-inflammatory cytokines TNF-α and IL-1β, in conjunction with ROS, such as the superoxide anion radical, are important peripheral and spinal hyperalgesic mediators and therefore represent relevant targets for analgesic drug development [13,14]. An analgesic role for vinpocetine is supported by its clinical utility in a wide range of neuroinflammatory human diseases, including multiple sclerosis [15], intracranial birth trauma-induced seizures [16], and chronic brain ischemia [17].

Data pertinent to the analgesic effect of vinpocetine show that it inhibits neuronal tetrodotoxin-resistant NaV1.8 sodium channel currents and sodium current-induced calcium influx [18,19], with intraperitoneal (i.p) administration of vinpocetine reducing acetic acid-induced visceral nociception, which was potentiated by muscarinic, adrenergic or opioid receptor blockade and dependent on adenosine receptors [20]. Vinpocetine also inhibits formalin-induced paw flinching, accompanied by inhibition of c-fos expression in the ipsilateral dorsal horn, when perineurally administered [21]. It has been also suggested that vinpocetine reduces neuropathic pain by blocking retrograde axoplasmic transport of nerve growth factor [21]. However, the peripheral and spinal effects of vinpocetine in inflammatory hyperalgesia and its mechanisms of action require investigation. Given the above literature, the present study aimed to investigate the effects of orally administered vinpocetine in a murine inflammatory pain model. In addition, we also assessed whether the analgesic effects of vinpocetine were related to peripheral and spinal inhibition of oxidative stress and cytokine production, as well as NF-κB activity.

## Material and Methods

### Animals

Male Swiss mice (25–30 g) from the Universidade Estadual de Londrina, Londrina, Paraná, Brazil, were used in this study. Mice were housed in standard clear plastic cages with free access to food and water with a light/dark cycle of 12/12h at a constant temperature of 21°C. All behavioral testing was performed between 9 a.m. and 5 p.m. in a temperature-controlled (21°C) room. Animal care and handling procedures were approved by the Ethics Committee of the Universidade Estadual de Londrina (process number 13278.2011.3). All efforts were made to minimize animal suffering and to reduce the number of animals used.

### Drugs and stimuli

Materials were obtained from the sources as follows: vinpocetine (Marjan Indústria & Comércio Ltda, São Paulo, Brazil), carrageenan (FMC Corp, Philadelphia, PA, United States), acetic acid and formaldehyde (Mallinckrodt Baker, S.A., Mexico, Mexico City), phenyl-*p*-benzoquinone (Sigma Chemical Company, St. Louis, MO).

### Experimental procedures

Mice received per oral (p.o.) treatment with vinpocetine (1, 3, 10, or 30 mg/kg) 1 h before the inflammatory stimulus. The dose of inflammatory stimuli was based on previous studies in our laboratory [22–25]. The writhing response was evaluated during the 20 min after i.p. injection of phenyl-p-benzoquinone (PBQ) (1890 μg/kg) or acetic acid 0.6% (10 mL/kg). The number of formalin (formalin 1.5%, 25 μL/paw)-induced flinches was evaluated during the 30 min after injection. Mechanical and thermal hyperalgesia was evaluated 1–9 h after carrageenan (100 μg, 25 μL, i.pl.), with neutrophil recruitment in paw skin indicated by myeloperoxidase (MPO) activity assay 9 h after carrageenan injection. IL-1β, TNF-α, total NF-κB and phosphorylated NF-κB levels were measured in the paw skin and spinal cord 3 h after carrageenan injection. The experimenters evaluating the recorded responses were blind as to specific treatments being assessed.

### Writing response tests

The PBQ and acetic acid-induced writhing models were performed as previously described [25]. PBQ (DMSO 2%, v/v in saline), acetic acid (0.6%, v/v in saline) or vehicle were injected into the peritoneal cavities of mice pre-treated with vinpocetine (1–30 mg/kg, p.o.). The intensity of overt pain-like behavior is expressed as the cumulative number of writhings over 20 minutes after stimulus injection.

### Formalin test

The number of paw flinches were determined between 0–30 minutes after 25 μL formalin i.pl. injection, as previously described [26]. The period was divided into intervals of 5 minutes, and clearly demonstrated the presence of the first (0–5 min) and second (15–30 min) phase, which are characteristic of the test. Results are presented as the number of flinches in the first and second phases.

### Mechanical hyperalgesia test

Mechanical hyperalgesia was measured by an electronic version of von Frey filaments [27]. The test consisted of evoking a hind paw reflex with a hand-held force transducer (electronic anesthesiometer; Insight, Ribeirao Preto, SP, Brazil). After the paw withdrawal, the intensity of the pressure was recorded automatically, with values being averaged across three measurements. Mice were tested before (basal) and after stimulus injection. The results are expressed as delta (Δ) withdrawal threshold (in g), calculated by subtracting the basal mean measurements from the mean measurements obtained at 1, 3, 5, 7, or 9 h after carrageenan i.pl. injection.

### Hot plate test

Mice were placed on a hot plate apparatus (IITC Life Science Inc. Woodland Hills, CA) maintained at 55°C. The reaction time was indicated by the time for the animal to jump or lick their paws. A maximum latency (cut-off) was set at 30 s to avoid tissue damage [28].

### MPO Activity

Myeloperoxidase (MPO) activity, an indicant of neutrophil recruitment to the paw skin, was evaluated by the MPO kinetic-colorimetric assay [29]. Samples were homogenized using a tissue-tearor (Biospec) in ice-cold K_2_HPO_4_ buffer (400 μL, 50 mM, pH 6.0) containing HTAB (0.5% weight/volume), and the homogenates were centrifuged (16100 *g* × 2 min × 4°C). The supernatants (30 μL) were mixed with K_2_HPO_4_ buffer (200 μL, 50 mM, pH 6.0) containing *o*-dianisidine dihydrochloride (0.0167%, w/v) and hydrogen peroxide (0.05%, v/v). The absorbance was determined after 5 min at 450 nm (Multiskan GO Microplate Spectrophotometer, Thermo Scientific, Vantaa, Finland). The results of MPO activity are expressed as the number of neutrophils per mg of tissue by using a standard curve of neutrophils (196–400000 cells).

### ABTS and FRAP assays

The ability of samples to resist oxidative damage was determined by their free radical scavenging (ABTS [2,2'-Azinobis-3-ethylbenzothiazoline 6-sulfonic acid] assay) and ferric reducing (FRAP assay) properties. The tests were adapted to a 96-well microplate format as previously described [30]. Plantar tissue samples were collected 3 h after carrageenan i.pl. injection (100 μg, 25 μL) and homogenized immediately in ice-cold KCl buffer (500 μL, 1.15% w/v). The homogenates were centrifuged (200 *g* × 10 min × 4°C), and the supernatants were used in both assays. Diluted ABTS solution (200 μL) was mixed with 10 μL of sample in each well. After 6 min of incubation at 25°C, the absorbance was measured at 730 nm. For FRAP assay, the supernatants (10 μL) were mixed with the freshly prepared FRAP reagent (150 μL). The reaction mixture was incubated at 37°C for 30 min, and the absorbance was measured at 595 nm (Multiskan GO Thermo Scientific). The results of ABTS and FRAP assays were equated against a standard Trolox curve (0.02–20 nmol).

### GSH levels measurement

Samples of paw skin and spinal cord were collected and maintained at −80°C for at least 48 h. The sample was homogenized with 200 μL of 0.02 M EDTA. The homogenate was mixed with 25 μL of trichloroacetic acid 50% and was homogenized three times over 15 min. The mixture was centrifuged (15 min x 1500 *g* x 4°C). The supernatant was added to 200 μL of 0.2 M TRIS buffer, pH 8.2, and 10 μL of 0.01M DTNB. After 5 min, the absorbance was measured at 412 nm (Multiskan GO, Thermo Scientific) against a blank reagent with no supernatant. A standard GSH curve was formed. The results are expressed as GSH per mg of protein [31].

### Superoxide anion production

The quantification of superoxide anion production in tissue homogenates (10 mg/mL in 1.15% KCl) was performed using the nitroblue tetrazolium (NBT) assay [32]. Briefly, 50 μL of the homogenate was incubated with 100 μL of NBT (1 mg/mL) in 96-well plates at 37°C for 1 h. The supernatant was then carefully removed and the reduced formazan solubilized by adding 120 μL of KOH 2M and 140 μL of DMSO. The NBT reduction was measured at 600 nm (Multiskan GO, Thermo Scientific). The tissue weight was used for data normalization.

### Cytokine measurement

Paw skin and spinal cord (L4-L6) samples were homogenized in 500 μL of buffer containing protease inhibitors, with IL-1β and TNF-α levels being determined as previously described by an enzyme-linked immunosorbent assay (ELISA) using eBioscience kits [31]. The results are expressed as picograms (pg) of cytokine/100 mg of tissue.

### NF-κB activity

The paw skin samples were collected and homogenized in ice-cold lysis buffer (Cell Signaling). The homogenates were centrifuged (200 *g* × 10 min × 4°C), with the supernatants used to assess the levels of phosphorylated and total NF-κB p65 subunit by ELISA using PathScan kits (Cell Signaling) according to the manufacturer’s directions. The results represent the sample ratio (total p65/phospho-p65) measured at 450 nm (Multiskan GO Thermo Scientific).

### Data analyses

Results are presented as means +/- SEM of measurements made on 6 mice per group per experiment and are representative of two independent experiments. Two-way ANOVA was used to compare the groups and doses at all times when the parameters were measured at different times after the stimulus injection. The analyzed factors were treatments, time, and time versus treatment interaction. One-way ANOVA followed by Tukey’s *t*-test was performed for each time-point. *P* < 0.05 was considered significant.

## Results

### Vinpocetine inhibits the acetic acid- and PBQ-induced writhing responses, as well as the formalin-induced paw flinches

**Fig 1:**
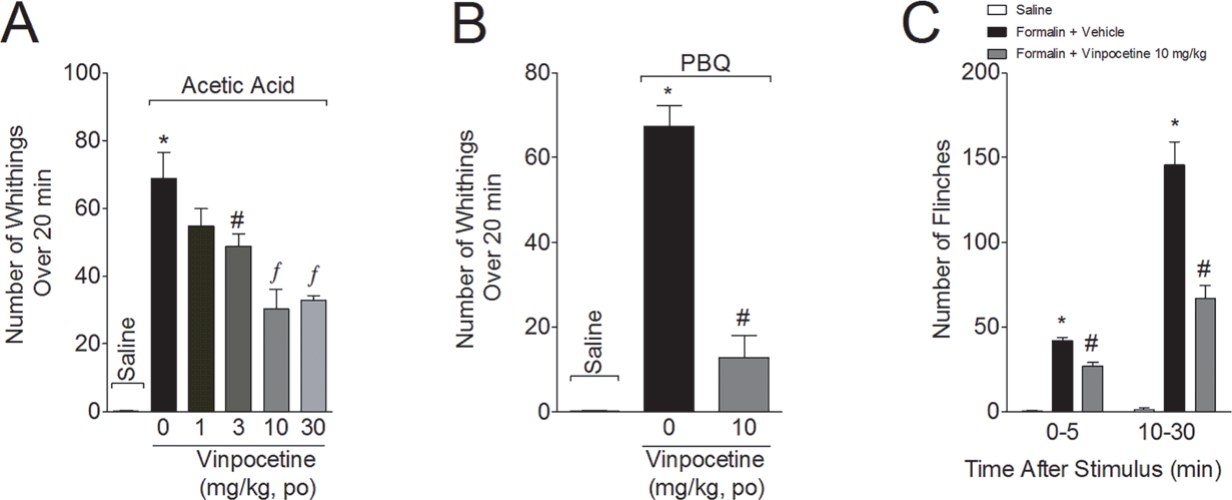
Vinpocetine inhibits overt pain-like behavior. Panel A: mice were treated with vinpocetine (1, 3, 10, and 30 mg/kg, p.o.) or vehicle (saline) 1 h before acetic acid i.p. injection (0.6% v/v, diluted in saline). Panels B-C: mice were treated with vinpocetine (30 mg/kg, p.o.) or vehicle (saline) 1 h before i.p. injection of phenyl-p-benzoquinone (PBQ, 1890 μg/kg in DMSO 2%, v/v, diluted in saline, panel B), or i.pl. injection of formalin (25 μL of 1.5% formalin, v/v in saline, panel C). The cumulative number of writhing was evaluated over 20 min (Panels A-B) and total number of paw flinches (Panel C) were evaluated over 30 min. Results are presented as means ± SEM of 6 mice per group per experiment, and are representative of 2 separate experiments. *P < 0.05 compared with the saline group; #*P* < 0.05 compared to the vehicle group; and ^*f*^ *P* < 0.05 compared to the vehicle group and the 1 mg/kg vinpocetine dose. One-way ANOVA followed by Tukey’s *t* test.

In the first series of experiments, the analgesic effect of vinpocetine was evaluated in acetic acid-, PBQ-, and formalin-induced overt pain-like behaviors. Mice were treated with vinpocetine (1, 3, 10, and 30 mg/kg p.o.) or vehicle (saline) 1 hour before acetic acid injection. Vinpocetine, at the doses of 3–30 mg/kg, inhibited the acetic acid-induced writhing response (Fig. 1A). At 1 mg/kg, vinpocetine showed no effect. A dose of 10 mg/kg of vinpocetine was selected for the following experiments, given that it achieved an inhibition of 56%, which was statistically similar to its inhibitory effect at 30 mg/kg. At a dose of 10 mg/kg, vinpocetine inhibited the PBQ-induced writhing response (81%) (Fig. 1B), and both the first (36%) and second phases (54%) of formalin-induced paw flinches (Fig. 1C).

### Vinpocetine inhibited carrageenan-induced hyperalgesia and MPO activity

**Fig 2:**
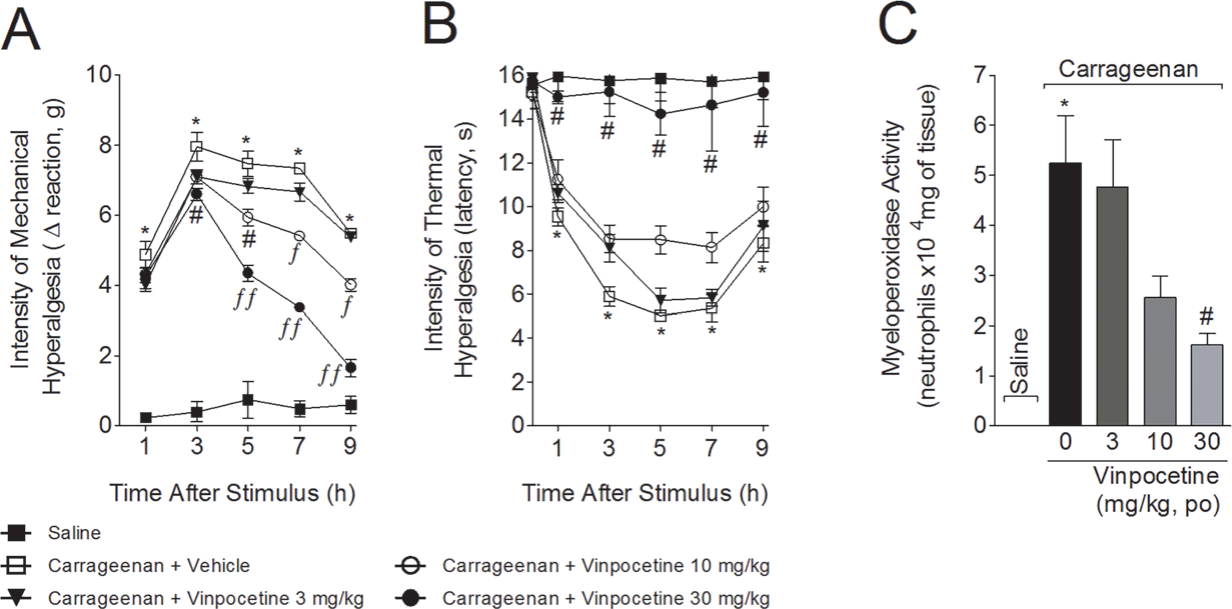
Vinpocetine inhibits carrageenan-induced hyperalgesia and myeloperoxidase (MPO) activity. Mice were treated with vinpocetine (3, 10 or 30 mg/kg, p.o.) or vehicle (saline) 1 h before carrageenan (100 μg, 25 μL) i.pl. injection. Mechanical (Panel A) and thermal (Panel B) hyperalgesia were assessed at indicated time points after carrageenan administration using the electronic von Frey and hot plate tests, respectively. Myeloperoxidase (MPO) activity was determined in samples collected 9 h after carrageenan injection. Results are presented as means ± SEM of 6 mice per group per experiment, and are representative of 2 separate experiments. *P < 0.05 compared to saline group; ^#^ *P* < 0.05 compared with vehicle group; ^*f*^ *P* < 0.05 compared with the dose of 3 mg/kg of vinpocetine; and ^*ff*^ *P* < 0.05 compared with the doses of 10 and 30 mg/kg of vinpocetine. One-way ANOVA followed by Tukey’s *t* test.

Mice were treated with vinpocetine (3, 10, or 30 mg/kg, p.o.) 1 h before carrageenan (100 μg, 25 μL) i.pl. injection, with mechanical (Fig. 2A) and thermal (Fig. 2B) hyperalgesia being evaluated at the indicated time points. After the last measurement (9 h), mice were euthanized and paw skin samples were collected to determine MPO activity (Fig. 2C). Carrageenan induced significant mechanical hyperalgesia, which was not affected by 3 mg/kg of vinpocetine (Fig. 2A). However, 10 mg/kg of vinpocetine significantly inhibited carrageenan-induced mechanical hyperalgesia at 5–9 h, with significant differences when compared to vinpocetine 3 mg/kg at 7 and 9 h. The dose of 30 mg/kg of vinpocetine inhibited carrageenan-induced mechanical hyperalgesia between 3 and 9 h (up to 78%), with significant differences compared to the lower doses of vinpocetine between 5 and 9 h. Vinpocetine at 30 mg/kg abolished carrageenan-induced thermal hyperalgesia (up to 100%) (Fig. 2B). Furthermore, 10 mg/kg of vinpocetine significantly inhibited carrageenan-induced thermal hyperalgesia at 5 h, being significantly more effective when compared to the lower dose of vinpocetine (Fig. 2B). MPO activity was reduced by vinpocetine at 30 mg/kg (69%) but not at lower vinpocetine doses (Fig. 2C). Given the results shown in Fig. 2, a vinpocetine dose of 30 mg/kg was selected for the following experiments.

### Vinpocetine inhibits carrageenan-induced reduction of ABTS free radical scavenging ability and ferric reducing antioxidant power (FRAP)

**Fig 3:**
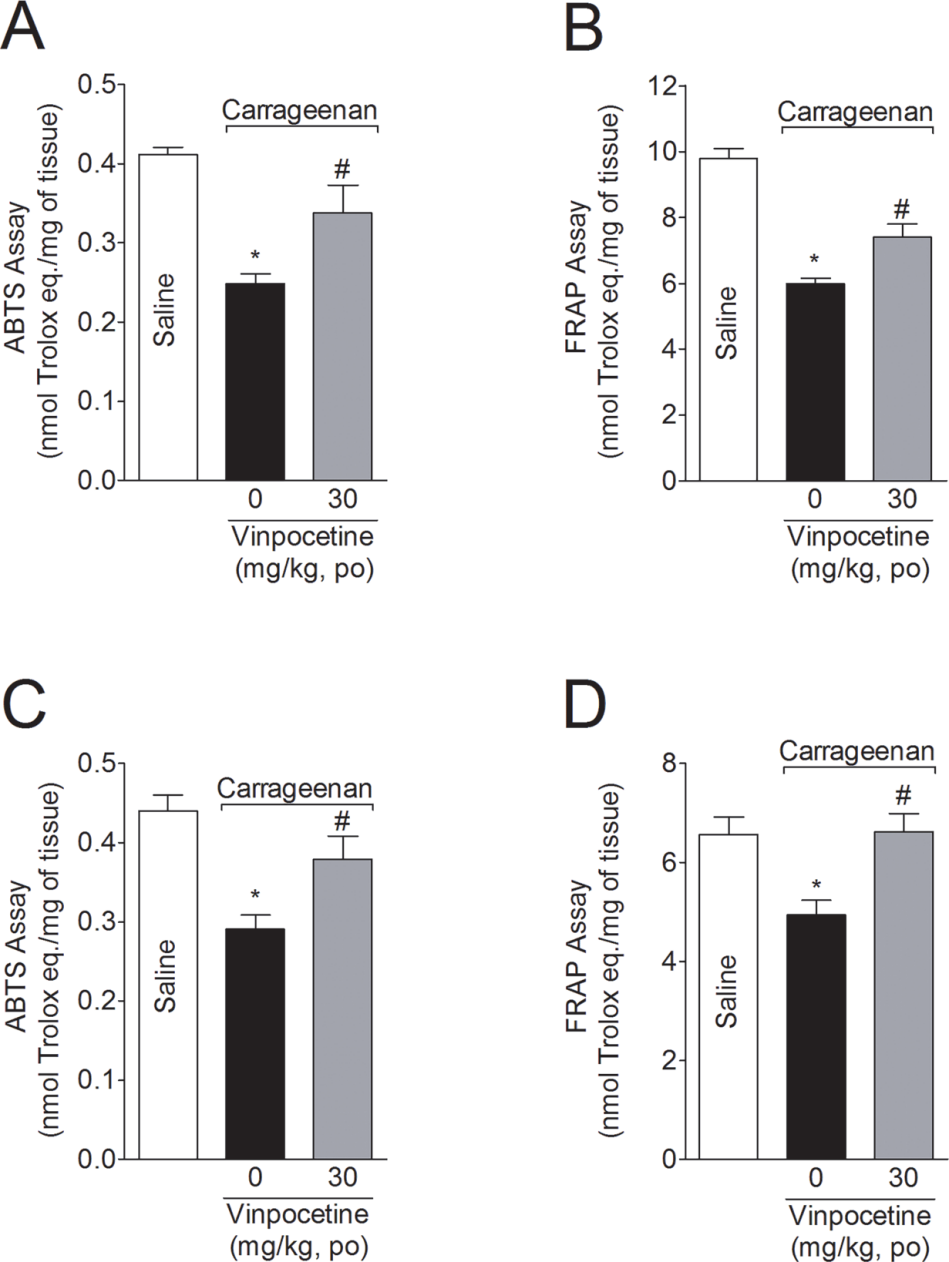
Vinpocetine inhibits carrageenan-induced decrease in antioxidant capacity. Mice were treated with vinpocetine (30 mg/kg, p.o.) or vehicle 1 h before carrageenan (100μg, 25 μL) i.pl. injection. Paw skin (Panels A and B) and spinal cord (Panels D and E) samples were collected 3 h after carrageenan injection for measurement of ABTS (Panels A and C) and FRAP (Panels B and D) assays. Results are presented as means ± SEM of 6 mice per group per experiment, and are representative of 2 separate experiments. *P < 0.05 compared with saline group; and ^#^ *P* < 0.05 compared with vehicle group. One-way ANOVA followed by Tukey’s *t* test.

Mice were treated with vinpocetine (30 mg/kg, p.o.) 1 h before injection with carrageenan (100 μg, 25 μL, i.pl). Mice were terminally anesthetized 3 h after the injection, with samples from the paw skin and spinal cord (L4-L6) being collected to evaluate the ABTS free radical scavenging ability and FRAP. Carrageenan reduced ABTS scavenging ability (Fig. 3A and 3C) and FRAP (Fig. 3B and 3D) in the paw skin and spinal cord samples. Vinpocetine treatment attenuated the carrageenan-induced decrease in ABTS scavenging ability (54 and 60%) and FRAP (37 and 100%) in paw skin and spinal cord samples, respectively.

### Treatment with vinpocetine attenuated the carrageenan-induced GSH depletion and superoxide anion production

**Fig 4:**
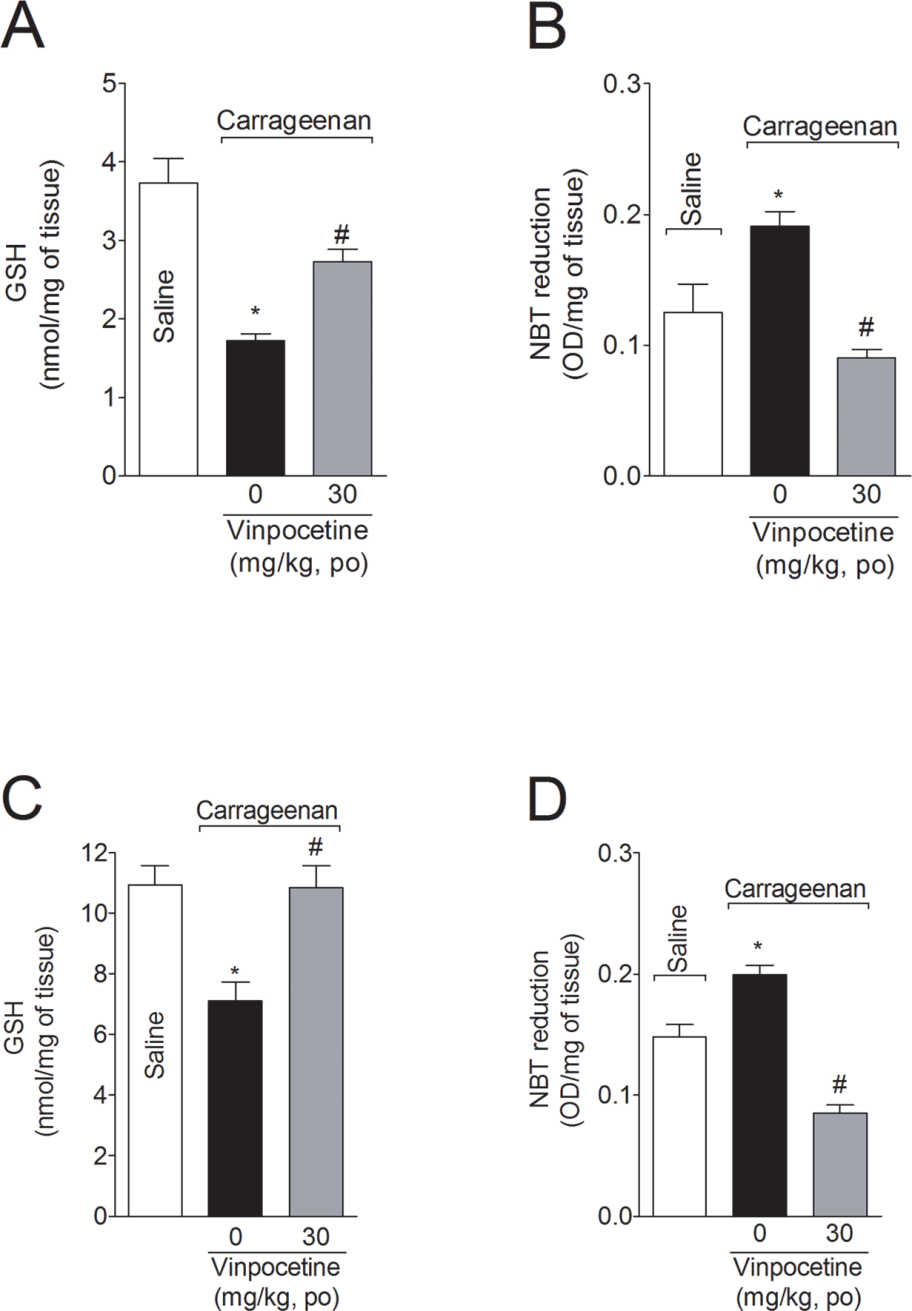
Vinpocetine inhibits carrageenan-induced depletion of reduced glutathione (GSH) levels and decreased nitroblue tetrazolium reduction (NBT) activity. Mice were treated with vinpocetine (30 mg/kg, p.o.) or vehicle 1 h before carrageenan i.pl. injection. Paw skin (Panels A and B) and spinal cord (Panels C and D) samples were collected 3 h after stimulus injection for the determination of GSH levels and NBT reduction. Results are presented as means ± SEM of 6 mice per group per experiment, and are representative of 2 separate experiments. *P < 0.05 compared with saline group; and ^#^ *P* < 0.05 compared with vehicle group. One-way ANOVA followed by Tukey’s *t* test.

Mice were treated with vinpocetine (30 mg/kg, p.o.) 1 h before i.pl. injection of carrageenan. Samples of cutaneous paw skin and spinal cord (L4-L6) were collected 3 h after carrageenan injection for GSH measurement (Fig. 4A and 4C, respectively). Carrageenan induced a significant GSH decrease in the paw skin (Fig. 4A) and spinal cord (Fig. 4B), which was inhibited by vinpocetine (99% and 50%, respectively). Carrageenan also induced superoxide anion production in the paw skin (Fig. 4B) and spinal cord (Fig. 4D), which were abolished by vinpocetine 30 mg/kg treatment.

### Vinpocetine inhibits carrageenan-induced IL-1β and TNF-α production in the paw skin and spinal cord

**Fig 5:**
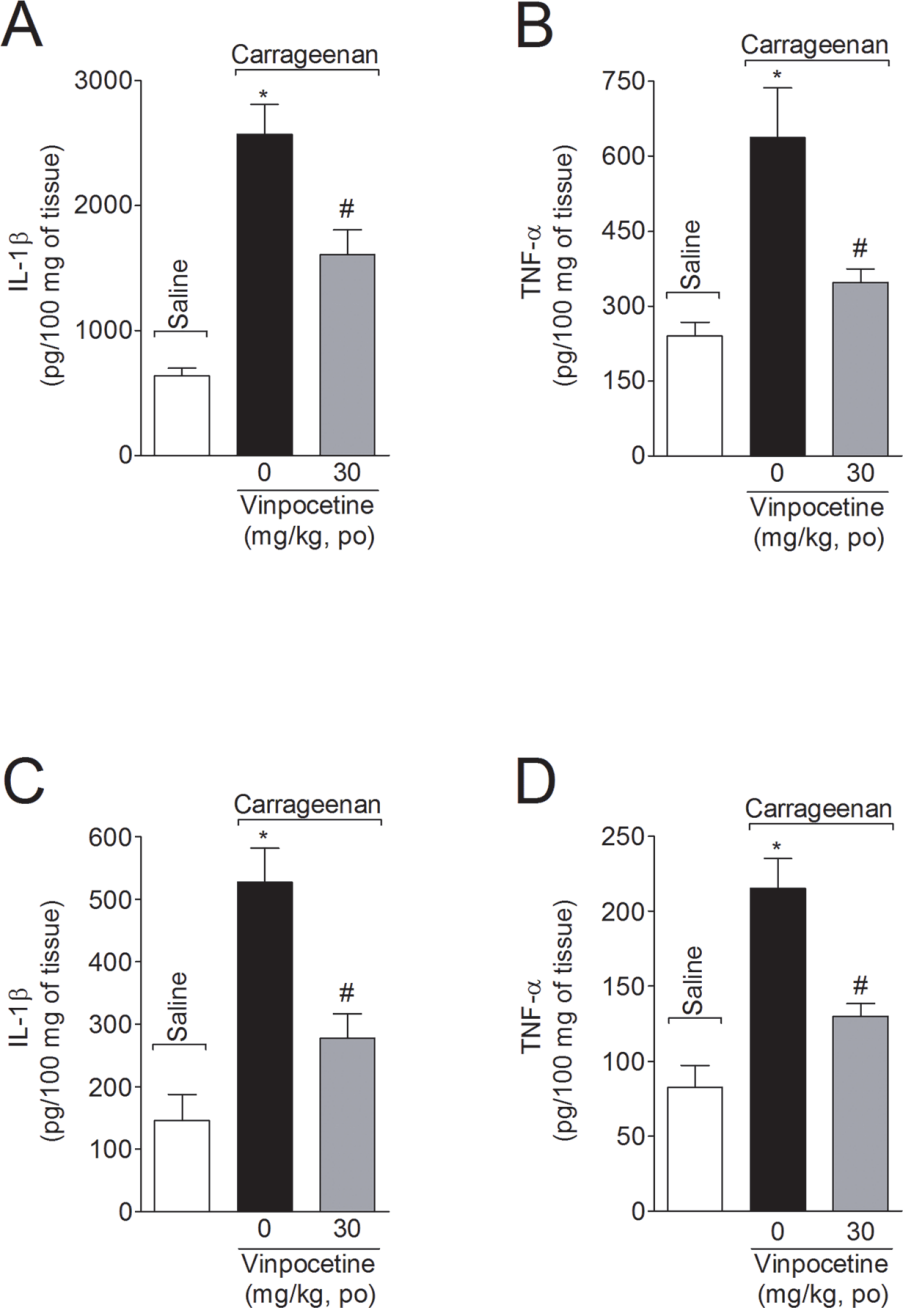
Vinpocetine inhibits carrageenan-induced IL-1β and TNF-α production. Mice were treated with vinpocetine (30 mg/kg, p.o.) or vehicle 1 h before carrageenan i.pl. injection. Paw skin (Panels A and B) and spinal cord (Panels C and D) samples were collected for the determination of IL-1β (Panels A and C) and TNF-α (Panels B and D) production by ELISA, respectively. Results are presented as means ± SEM of 6 mice per group per experiment, and are representative of 2 separate experiments. *P < 0.05 compared with saline group; and ^#^ *P* < 0.05 compared with vehicle group. One-way ANOVA followed by Tukey’s *t* test.

Mice were treated with vinpocetine (30 mg/kg, p.o.) 1h before carrageenan i.pl. injection. Samples from the paw skin and spinal cord tissue (L4-L6) were collected 3 h after carrageenan injection for IL-1β and TNF-α measurement (Fig. 5). Carrageenan significantly increased IL-1β and TNF-α levels in paw skin (Fig. 5A and 5B, respectively) and spinal cord (Fig. 5C and 5D, respectively), which were attenuated by vinpocetine treatment (49% and 73%, and 65% and 64%, respectively).

### Vinpocetine attenuates peripheral and spinal NF-κB activation induced by carrageenan

**Fig 6:**
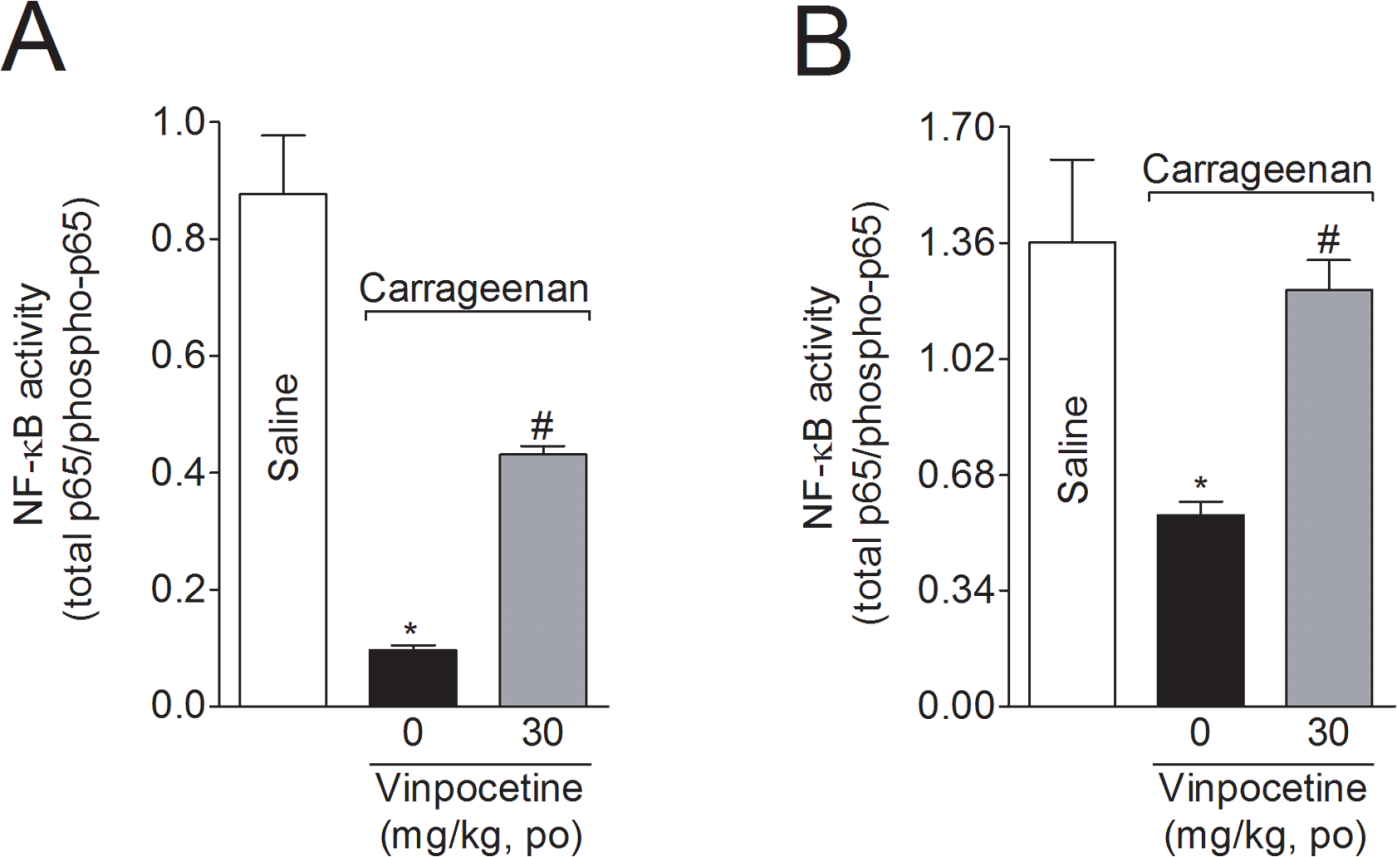
Vinpocetine inhibits carrageenan-induced NF-κB activation. Mice were treated with vinpocetine (30 mg/kg, p.o.) or vehicle 1 h before carrageenan i.pl. injection. Paw skin (Panel A) and spinal cord (Panel B) samples were collected 3 h after stimulus injection in lysis buffer, and the NF-κB activation was measured by ELISA, as a ratio of total NF-κB/phosphorylated NF-κB. Results are presented as means ± SEM of 6 mice per group per experiment, and are representative of 2 separate experiments. *P < 0.05 compared with saline group; and ^#^ *P*< 0.05 compared with vehicle group. One-way ANOVA followed by Tukey’s *t* test.

The levels of total and phosphorylated NF-κB p65 subunits were evaluated in samples from the paw skin and spinal cord (L4-L6). Mice were treated with vinpocetine (30 mg/kg) 1 h before carrageenan i.pl. injection, with the samples being collected 3 h after injection. Vinpocetine (Fig. 5) attenuated carrageenan-induced NF-κB activation in the paw skin (Fig. 6A, 43%) and spinal cord (Fig. 6B, 83%) samples.

## Discussion

Increased pain sensitivity is a common feature of the inflammatory response and occurs after tissue injury. The peripheral sensitization is triggered by NF-κB-related pro-inflammatory mediators, including the cytokines TNF-α and IL-1β [14,33,34], as well as ROS such as the superoxide anion radical [35]. In addition to their role in the inflammatory response, these mediators can act directly on their receptors or targets expressed by peripheral terminals of nociceptors to reduce pain thresholds, leading to inflammatory pain and hyperalgesia [36,37]. Moreover, they induce the recruitment of neutrophils, leading to the further enhancement of hyperalgesic mediators, such as prostaglandin E_2_ (PGE_2_) and the superoxide anion [33,35]. Consequently, inhibiting the production of such hyperalgesic mediators is an important pain control target [14,34]. It is important to note that in a number of diseases inflammatory hyperalgesia is the major symptom responsible for reducing quality of life [38,39], which is often further reduced by the side effects occurring from the chronic use of corticosteroids and non-steroid anti-inflammatory drugs [2,3]. In this study, we described the anti-hyperalgesic effects of vinpocetine, showing that its mechanism of action targets peripheral and spinal cord oxidative stress, TNF-α, IL-1β and NF-κB activation.

Vinpocetine is a safe nootropic agent that is widely used to improve memory and to treat neurological diseases. Vinpocetine has a low oral bioavailability, achieving about 0.7% absorption in humans [40], although its absorption may increase to 60–100% in non-fasted individuals [41]. Radio-labeled vinpocetine (10 mg tablet) administered orally to humans appears in the blood and brain shortly after administration and increases continuously for at least 120 and 100 min, respectively [40]. These being the maximal periods investigated in each tissue. [40]. Therefore, orally administrated vinpocetine is rapidly distributed throughout the entire body explaining its therapeutic use and commercialization. Preclinical studies using acetic acid- and formalin-induced overt pain-like behaviors shows vinpocetine to exert analgesic effects when administered via i.p or perineural routes, respectively [20,21]. Our results support such research, showing that vinpocetine exhibits analgesic effects in acetic acid-, PBQ- and formalin-induced overt pain-like behaviors, even when administered orally, thereby confirming its applicability as an orally active analgesic in such models. In fact, the potential therapeutic importance of vinpocetine has generated a large number of studies that have attempted to improve its absorption [42], thereby allowing a reduced dose for therapeutic effect and/or increased efficacy. Importantly, these animal models, except the first phase of formalin test, depend on cytokine production to induce inflammation and pain [25,43], suggesting that inflammatory pain attenuation by vinpocetine depends, at least in part, on decreasing the production of such mediators.

Recent work shows vinpocetine to afford protection in numerous inflammatory conditions, including atherosclerosis [44,45], lung inflammation [9], macular degeneration [46] and cerebral ischemia–reperfusion injury [47]. In these studies, the vinpocetine anti-inflammatory effect was at least partly driven by its attenuation of NF-κB activity and its inflammatory products, such as TNF-α and IL-1β. Carrageenan and lipopolysaccharide (LPS) from gram-negative bacteria activate TLR4/MyD88 signaling, thereby triggering NF-κB activation and the cytokines, including TNF-α and IL-1β, which are responsible for pain and inflammation [48–50]. Targeting TLR4/MyD88, NF-κB, TNF-α and IL-1β reduces carrageenan- and LPS-induced hyperalgesia [14,33,51]. Therefore, we reason that the attenuation of NF-κB activity, including by decreasing TNF-α and IL-1β production, is an important determinant of vinpocetine's analgesic effects.

NF-κB heterodimer p50/p65 activates the transcription of a large number of genes, many of which are key mediators of the inflammatory response [52–54]. Inhibiting NF-κB signaling impairs neutrophil recruitment during acute inflammation by reducing the expression of NF-κB target genes, including pro-inflammatory cytokines and chemokines [55–57]. TNF-α and IL-1β are crucial mediators in the recruitment of neutrophils after tissue injury by directly chemoattracting neutrophils and activating resident inflammatory cells, which further amplifies the production of chemotactic molecules [56–59]. Neutrophils, in turn, play an important role in carrageenan-induced hyperalgesia as these cells provide an important source of the superoxide anion and other hyperalgesic mediators, such as PGE_2_ [33,60,61]. In this study, we found that vinpocetine reduced neutrophil recruitment, as assessed by MPO activity. Neutrophils produce large amounts of the superoxide anion, thereby contributing to many of the deleterious effects of inflammation, such as oxidative stress, tissue injury and hyperalgesia [33,60,61]. In agreement with this, we showed vinpocetine to also inhibit the carrageenan-induced peripheral decrease in antioxidant capacity and GSH levels. Previous work, in a rat demyelination model, showed vinpocetine to reduce oxidative stress [12], although, the association of oxidative stress with increased pro-inflammatory cytokines was not addressed in this study. Considering the dependency of carrageenan-induced hyperalgesia on not only TNF-α and IL-1β production [33], but also oxidative stress [35,62], and the peripheral inhibition of these cytokines and oxidative stress by vinpocetine treatment reported here, our results serve to better integrate previous work that separately described the analgesic, anti-inflammatory and antioxidant effects of vinpocetine [9,11,12,20,21,45–47].

Inflammation-induced peripheral nociceptor sensitization leads to increases in nociceptor inputs and in nociceptive stimuli transmission. This enhanced afferent activity, in turn, induces long-lasting increases in the excitability of spinal cord neurons and contributes to inflammatory pain hypersensitivity [63]. This central sensitization shares some mechanisms with the peripheral sensitization process. At the level of the spinal cord, as well as in dorsal root ganglion, glial cells act as sentinels of neuronal activity and damage. Glial cells produce inflammatory mediators in response to raised glutamate, as occurs during increased neurotransmission. Among these mediators, TNF-α, IL-1β and the superoxide anion act synergistically to sensitize nociceptive neurons [63,64]. In line with this, we found that vinpocetine also attenuated NF-κB activation, as well as TNF-α, IL-1β and oxidative stress at the spinal cord level, which may represent an important anti-hyperalgesic mechanism in the spinal cord. In line with this hypothesis, vinpocetine can inhibit glutamate signaling [65] as well as the calcium and sodium channel-mediated release of glutamate from nerve endings [18,66]. Vinpocetine also affords neuroprotection by reducing NF-κB expression, and IL-1β and TNF-α production, by microglia cells [9,11,18]. The central anti-inflammatory effect of vinpocetine was further shown by its decrease in hippocampal TNF-α and IL-1β production in a preclinical study of the pro-convulsive agents, 4-aminopyridine, pentylenetetrazole and pilocarpine, where vinpocetine inhibited cytokine production induced by increased neuronal activity [67].

The antioxidant effects of vinpocetine described in cerebral isolated nerve endings are similar to those of α-tocopherol, a classical antioxidant [10]. Considering its high permeability through the blood-brain barrier [68], it is possible that vinpocetine is acting at the spinal level to inhibit NF-κB activation, pro-inflammatory mediator production and oxidative stress, thereby preventing the resulting central sensitization. Alternatively, given that peripheral carrageenan injection induces CX_3_CL1 production by neurons, which activates satellite glial cells to release TNF-α and IL-β around the dorsal root ganglion that contributes to nociceptor sensitization [69], the peripheral inhibition of inflammation by vinpocetine may result in reduced activation (oxidative stress, cytokine production, NF-κB activation) in the spinal cord.

Additionally, vinpocetine has direct neuronal effects that could also reduce the nociceptive inputs, including blocking the retrograde axoplasmic transport of nerve growth factor, which is proposed as its analgesic mechanism of action in neuropathic pain [21]. Vinpocetine also blocks NaV1.8 sodium currents [19,70–73], being another means whereby it may act directly to inhibit nociceptor activation. In fact, we observed that vinpocetine reduced the first phase (0–5 min) of the formalin test, which is considered a neurogenic phase [43,74], with the second phase depending on hyperalgesic inflammatory mediators. One important step during inflammation-triggered hyperalgesia is increased intracellular calcium levels in nociceptors [75], which vinpocetine may achieve by reducing sodium currents-induced intracellular calcium level increases, as shown by previous data in rat hippocampal pyramidal cells [76]. Moreover, systemic treatment with vinpocetine delays the reaction time of naïve mice in the hot plate test [20] and reduces oxidative stress levels in rat brain [12], suggesting that supraspinal mechanisms are also involved. Such data is in concordance with the more pronounced effects of vinpocetine to reduce thermal hyperalgesia, as reported here.

Vinpocetine is also known as a phosphodiasterase-1 (PDE-1) inhibitor, which degrades cyclic guanosine monophosphate (cGMP) and cyclic adenosine monophosphate (cAMP) [77]. Increasing cGMP levels is an analgesic mechanism of nitric oxide donors, as well as of analgesics in clinical use [78,79]. TNF-α and IL-1β induce PGE_2_ production, which is ultimately responsible for nociceptor sensitization and hyperalgesia development in the carrageenan model [80,81]. PGE_2_ activates EP3 and EP4 receptors increasing cAMP and PKA to induce hyperalgesia [82]. Moreover, PKCε is also activated by increased cAMP levels in nociceptive neurons and contributes to the hyperalgesia induced [83]. As such, increasing neuronal cAMP levels would not be likely to contribute to the analgesic effects of vinpocetine. On the other hand, increasing cAMP in tissue resident cells such as macrophages can reduce inflammation by, for instance, inducing receptor shedding [84] and inhibiting cytokine production [85]. Additionally, it must be pointed out that vinpocetine also directly activates the large-conductance calcium-activated potassium channels (BK_Ca_) in neurons and neuroendocrine cells, which contribute to membrane hyperpolarization and thus also represents a possible analgesic mechanism of vinpocetine [86]. As such, it remains to be determined as to the contribution of PDE-1 and BK_Ca_ channels to the analgesic effect of vinpocetine.

It is noteworthy that vinpocetine inhibits TNF-α- and LPS-induced inflammatory mediators and NF-κB activation by targeting IκB kinase (IKK) activity in a variety of cell lines, as well as in models of lung inflammation [9]. In this same study, IC86340, a highly selective PDE-1 inhibitor did not affect TNF-α-induced NF-κB activation or IKK activity. Furthermore, TNF-α-induced IKK activity, IκB phosphorilation and IκB degradation were not affected by nifedipine (Ca2+ channel blocker), EGTA (extracellular Ca2+ chelator), 1,2-bis(o-Aminophenoxy)ethane-N,N,N′,N′-tetraacetic Acid Tetra(acetoxymethyl) Ester (BAPTA/AM; an intracellular Ca2+ chelator) and tetrotodotoxin (Na+ channel inhibitor) in rat vascular smooth muscle cells [9]. Additionally, although there is evidence that vinpocetine inhibits PDE-4, it did not inhibit PDE-4 as effectively as rolipram, a classical PDE-4 inhibitor with anti-inflammatory effects [9,87]. Therefore, despite reports indicating that vinpocetine inhibits PDE-1, PDE-4, Ca2+ and Na+ channels [18,19,37,66,71–73,88], none of these mechanisms participated in the anti-inflammatory effects of vinpocetine that involve targeting IKK activity that consequently attenuates NF-κB activity, suggesting that there may be other novel vinpocetine mechanism(s) of action [9].

Finally, it is important to highlight that vinpocetine exhibits gastric protective [89] and antidepressant [20,90] effects, suggesting that vinpocetine is a highly promising and safe candidate treatment for the clinical management of inflammation and pain, thereby improving patients' quality of life.

## Conclusions

Vinpocetine inhibited inflammatory pain by targeting NF-κB activation and the NF-κB-driven transcriptional consequences, including cytokine production and oxidative stress, both peripherally in the paw skin and centrally in the spinal cord. Therefore, given that vinpocetine is already in clinical use as a nootropic drug with a good safety profile, it will be important to further investigate its utility and mechanism(s) of action as an analgesic.
